# Supplementary material for: Modelling Skylarks (Alauda arvensis) to Predict Impacts of Changes in Land Management and Policy: Development and Testing of an Agent-Based Model
Source: PLoS One. 2013 Jun 6;8(6):e65803. doi: 10.1371/journal.pone.0065803 (PMC3675089; doi:10.1371/journal.pone.0065803)
Supplement: Supporting Information S4 — The skylark ODdox as a zipped archive. (ZIP) [file pone.0065803.s004.zip › Skylark_ODdox/class_skylark___clutch.html]

ALMaSS Skylark ODdox: Skylark\_Clutch Class Reference


ALMaSS Skylark ODdox
 2.0


- Main Page
- Related Pages
- Classes
- Files

- Class List
- Class Index
- Class Hierarchy
- Class Members

Public Member Functions |
Public Attributes |
Protected Member Functions |
Protected Attributes

Skylark\_Clutch Class Reference

`#include <skylarks_all.h>`

List of all members.

|  |  |
| --- | --- |
| Public Member Functions | |
| void | AddEgg () |
| virtual void | BeginStep (void) |
| virtual void | EndStep (void) |
| void | OnMumGone () |
|  | Skylark\_Clutch (Skylark\_Female \*Mum, SkTerritories \*Terrs, Landscape \*L, int NoEggs, int x, int y, int mh, Skylark\_Population\_Manager \*SPM) |
| void | StartDeveloping () |
| virtual void | Step (void) |
| Public Member Functions inherited from Skylark\_Base | |
| void | AddStriglingMort (int lifestage) |
| bool | InSquare (rectangle R) |
| virtual double | On\_FoodSupply (double) |
|  | Skylark\_Base (int x, int y, SkTerritories \*Terrs, Landscape \*L, Skylark\_Population\_Manager \*SPM, int bx, int by, int mh) |
| virtual int | WhatState () |
| Public Member Functions inherited from TAnimal | |
| void | CheckManagement (void) |
| void | CheckManagementXY (int x, int y) |
| virtual void | CopyMyself () |
| virtual void | Dying () |
| virtual void | KillThis () |
| void | SetX (int a\_x) |
| void | SetY (int a\_y) |
| int | Supply\_m\_Location\_x () |
| int | Supply\_m\_Location\_y () |
| unsigned | SupplyFarmOwnerRef () |
| int | SupplyPolygonRef () |
| AnimalPosition | SupplyPosition () |
|  | TAnimal (int x, int y, Landscape \*L) |
| Public Member Functions inherited from TALMaSSObject | |
| void | OnArrayBoundsError () |
|  | TALMaSSObject () |
| virtual | ~TALMaSSObject () |

|  |  |
| --- | --- |
| Public Attributes | |
| int | Clutch\_Size |
| Skylark\_Female \* | Mother |
| Public Attributes inherited from Skylark\_Base | |
| int | Age |
| int | m\_Born\_x |
| int | m\_Born\_y |
| TTypesOfSkState | m\_CurrentSkState |
| int | m\_MyHome |
|  | The vegetation type where the skylark was born. |
| Skylark\_Population\_Manager \* | m\_OurPopulationManager |
| SkTerritories \* | m\_OurTerritories |
| double | m\_pcide\_conc |
| double | m\_pesticide\_accumulation |
| double | m\_Size |

|  |  |
| --- | --- |
| Protected Member Functions | |
| virtual bool | OnFarmEvent (FarmToDo event) |
| int | st\_Developing () |
| void | st\_Dying () |
| int | st\_Hatching () |
| Protected Member Functions inherited from Skylark\_Base | |
| bool | DailyMortality (int mort) |
| virtual void | PesticideResponse () |
| Protected Member Functions inherited from TAnimal | |
| void | CorrectWrapRound (void) |
|  | Corrects wrap around co-ordinate problems. |

|  |  |
| --- | --- |
| Protected Attributes | |
| int | m\_baddays |
| int | MinDegrees |

---

## Constructor & Destructor Documentation

|  |  |  |  |
| --- | --- | --- | --- |
| Skylark\_Clutch::Skylark\_Clutch | ( | Skylark\_Female \* | *Mum*, |
|  |  | SkTerritories \* | *Terrs*, |
|  |  | Landscape \* | *L*, |
|  |  | int | *NoEggs*, |
|  |  | int | *x*, |
|  |  | int | *y*, |
|  |  | int | *mh*, |
|  |  | Skylark\_Population\_Manager \* | *SPM* |
|  | ) |  |  |

References Skylark\_Base::Age, Clutch\_Size, m\_baddays, MinDegrees, and Mother.

: Skylark\_Base( x, y, Terrs, L, SPM, x, y, m\_MyHome ) {

Age = 0;

Clutch\_Size = NoEggs;

MinDegrees = 0;

Mother = Mum;

m\_baddays = 0;

}

---

## Member Function Documentation

|  |  |  |  |  |
| --- | --- | --- | --- | --- |
| void Skylark\_Clutch::AddEgg | ( |  | ) |  |

inline

Referenced by Skylark\_Female::st\_Laying().

{

Clutch\_Size++;

}

|  |  |  |  |  |  |
| --- | --- | --- | --- | --- | --- |
| void Skylark\_Clutch::BeginStep | ( | void |  | ) |  |

virtual

Reimplemented from TAnimal.

References TAnimal::CheckManagement().

{

CheckManagement();

}

|  |  |  |  |  |  |
| --- | --- | --- | --- | --- | --- |
| void Skylark\_Clutch::EndStep | ( | void |  | ) |  |

virtual

Reimplemented from TAnimal.

References TALMaSSObject::CurrentStateNo, Calendar::Date(), g\_date, Skylark\_Base::m\_CurrentSkState, st\_Developing(), st\_Dying(), st\_Hatching(), TALMaSSObject::StepDone, toss\_CDying, toss\_Destroy, toss\_Developing, toss\_Hatching, toss\_Initiation, and Landscape::Warn().

{

#ifdef \_\_CJTDebug\_5

if ( IsAlive() != 0x0DEADC0DE ) DEADCODEError();

#endif

if ( m\_CurrentSkState == toss\_Destroy ) return;

switch ( m\_CurrentSkState ) {

case toss\_Initiation:

break;

case toss\_Developing: // Develop

switch ( st\_Developing() ) {

case 2:

#ifdef TEST\_ISSUE\_DEATH\_WARRANT

printf( "Skylark\_Clutch::EndStep() st\_Developing() %d\n", ( int )g\_date->Date() );

#endif

m\_CurrentSkState = toss\_CDying; // die

break;

case 1:

m\_CurrentSkState = toss\_Hatching; // hatch

break;

case 0:

break;

}

break;

case toss\_Hatching: // Hatching

switch ( st\_Hatching() ) {

case 0:

#ifdef TEST\_ISSUE\_DEATH\_WARRANT

printf( "Skylark\_Clutch::EndStep() st\_Hatching() %d\n", ( int )g\_date->Date() );

#endif

m\_CurrentSkState = toss\_CDying;

break;

case 1: // Successful hatch

m\_CurrentSkState = toss\_Destroy; // will kill it at end of step!

CurrentStateNo = -1;

StepDone = true; // otherwise it will loop forever

break;

}

break;

case toss\_CDying: // Dying

st\_Dying();

StepDone = true;

break;

default:

char errornum[ 20 ];

sprintf( errornum, "%d", m\_CurrentSkState );

g\_land->Warn( "Skylark\_Clutch::EndStep(): Unknown state: ", errornum );

exit( 0 );

break;

}

}

|  |  |  |  |  |  |
| --- | --- | --- | --- | --- | --- |
| bool Skylark\_Clutch::OnFarmEvent | ( | FarmToDo | *event* | ) |  |

protectedvirtual

Reimplemented from TAnimal.

References Skylark\_Base::AddStriglingMort(), autumn\_harrow, autumn\_or\_spring\_plough, autumn\_plough, autumn\_roll, autumn\_sow, burn\_straw\_stubble, cattle\_out, cattle\_out\_low, cut\_to\_hay, cut\_to\_silage, cut\_weeds, Calendar::Date(), deep\_ploughing, Landscape::EventtypeToString(), fa\_ammoniumsulphate, fa\_greenmanure, fa\_manure, fa\_npk, fa\_pk, fa\_sludge, fa\_slurry, fp\_greenmanure, fp\_liquidNH3, fp\_manganesesulphate, fp\_manure, fp\_npk, fp\_npks, fp\_pk, fp\_sludge, fp\_slurry, fungicide\_treat, g\_date, glyphosate, growth\_regulator, harvest, hay\_bailing, hay\_turning, herbicide\_treat, hilling\_up, insecticide\_treat, Skylark\_Base::m\_CurrentSkState, TAnimal::m\_Location\_x, TAnimal::m\_Location\_y, TAnimal::m\_OurLandscape, molluscicide, mow, pigs\_out, product\_treat, random(), row\_cultivation, sleep\_all\_day, spring\_harrow, spring\_plough, spring\_roll, spring\_sow, straw\_chopping, strigling, strigling\_sow, stubble\_harrowing, Landscape::SupplyGrazingPressure(), swathing, syninsecticide\_treat, toss\_CDying, trial\_insecticidetreat, CfgInt::value(), Landscape::Warn(), water, and winter\_plough.

{

switch ( event ) {

case sleep\_all\_day:

break;

case autumn\_plough:

case autumn\_harrow:

case autumn\_roll:

case autumn\_sow:

case winter\_plough:

case deep\_ploughing:

case spring\_plough:

case spring\_harrow:

case spring\_roll:

case spring\_sow:

case fp\_liquidNH3:

case fp\_greenmanure:

case row\_cultivation:

case hilling\_up:

case harvest:

case cut\_to\_hay:

case cut\_to\_silage:

case straw\_chopping:

case hay\_turning:

case autumn\_or\_spring\_plough:

case burn\_straw\_stubble:

case mow:

#ifdef TEST\_ISSUE\_DEATH\_WARRANT

printf( "Skylark\_Clutch::OnFarmEvent() : %d : %d\n", event, ( int )g\_date->Date() );

#endif

m\_CurrentSkState = toss\_CDying;

break;

case fp\_npks:

case fp\_npk:

case fp\_pk:

case fp\_manganesesulphate:

case fp\_manure:

case fp\_sludge:

case fa\_npk:

case fa\_pk:

case fa\_ammoniumsulphate:

case fa\_manure:

case fa\_greenmanure:

case fa\_sludge:

case herbicide\_treat:

case growth\_regulator:

case fungicide\_treat:

case insecticide\_treat:

case trial\_insecticidetreat:

case syninsecticide\_treat:

if ( random( 1000 ) < cfg\_insecticide\_direct\_mortE.value() ) {

#ifdef TEST\_ISSUE\_DEATH\_WARRANT

printf( "Skylark\_Female::OnFarmEvent() : %d : %d\n", event, ( int )g\_date->Date() );

#endif

m\_CurrentSkState = toss\_CDying;

}

break;

case molluscicide:

case water:

case hay\_bailing:

case stubble\_harrowing:

case cut\_weeds:

break;

case fp\_slurry:

case fa\_slurry:

case swathing:

if ( random( 100 ) < 2 ) {

#ifdef TEST\_ISSUE\_DEATH\_WARRANT

printf( "Skylark\_Clutch::OnFarmEvent() : %d : %d\n", event, ( int )g\_date->Date() );

#endif

m\_CurrentSkState = toss\_CDying;

}

break;

case strigling:

#ifndef \_\_NoStriglingEffect

if ( random( 100 ) < cfg\_strigling\_clutch.value() ) {

#ifdef TEST\_ISSUE\_DEATH\_WARRANT

printf( "Skylark\_Clutch::OnFarmEvent() : %d : %d\n", event, ( int )g\_date->Date() );

#endif

m\_CurrentSkState = toss\_CDying;

AddStriglingMort( 1 );

}

#endif

break;

case cattle\_out:

if ( m\_OurLandscape->SupplyGrazingPressure( m\_Location\_x, m\_Location\_y ) )

if ( random( 1000 ) < 20 ) { // was 20

#ifdef TEST\_ISSUE\_DEATH\_WARRANT

printf( "Skylark\_Clutch::OnFarmEvent() : %d : %d\n", event, ( int )g\_date->Date() );

#endif

m\_CurrentSkState = toss\_CDying;

}

break;

case cattle\_out\_low:

if ( m\_OurLandscape->SupplyGrazingPressure( m\_Location\_x, m\_Location\_y ) )

if ( random( 1000 ) < 5 ) { //was 5

#ifdef TEST\_ISSUE\_DEATH\_WARRANT

printf( "Skylark\_Clutch::OnFarmEvent() : %d : %d\n", event, ( int )g\_date->Date() );

#endif

m\_CurrentSkState = toss\_CDying;

}

break;

case pigs\_out:

#ifndef \_\_NoPigsOutEffect

if ( random( 100 ) < 20 ) {

#ifdef TEST\_ISSUE\_DEATH\_WARRANT

printf( "Skylark\_Clutch::OnFarmEvent() : %d : %d\n", event, ( int )g\_date->Date() );

#endif

m\_CurrentSkState = toss\_CDying;

}

#endif

break;

case strigling\_sow:

#ifndef \_\_NoStriglingEffect

if ( random( 100 ) < cfg\_strigling\_clutch.value() ) {

#ifdef TEST\_ISSUE\_DEATH\_WARRANT

printf( "Skylark\_Clutch::OnFarmEvent() : %d : %d\n", event, ( int )g\_date->Date() );

#endif

m\_CurrentSkState = toss\_CDying;

AddStriglingMort( 1 );

}

#endif

break;

case product\_treat:

break;

case glyphosate:

break;

default:

g\_land->Warn( "Skylark\_Clutch::OnFarmEvent(): Unknown event type:", m\_OurLandscape->EventtypeToString( event ) );

exit( 1 );

}

if ( m\_CurrentSkState == toss\_CDying ) return true; else

return false;

}

|  |  |  |  |  |  |
| --- | --- | --- | --- | --- | --- |
| void Skylark\_Clutch::OnMumGone | ( | void |  | ) |  |

References TALMaSSObject::CurrentStateNo, Calendar::Date(), g\_date, Skylark\_Base::m\_CurrentSkState, Mother, and toss\_Destroy.

Referenced by Skylark\_Female::OnMateDying(), Skylark\_Female::OnMateHomeless(), Skylark\_Female::st\_Dying(), Skylark\_Female::st\_GivingUpTerritory(), Skylark\_Female::st\_StartingNewBrood(), Skylark\_Female::st\_StoppingBreeding(), and Skylark\_Female::Step().

{

#ifdef \_\_CJTDebug\_5

if ( IsAlive() != 0x0DEADC0DE )

DEADCODEError();

#endif

// the clutch cannot survive so go directly to die and do not pass go

Mother = NULL;

#ifdef TEST\_ISSUE\_DEATH\_WARRANT

printf( "Skylark\_Clutch::OnMumGone() %d\n", ( int )g\_date->Date() );

#endif

m\_CurrentSkState = toss\_Destroy; // will kill it at end of step!

CurrentStateNo = -1;

}

|  |  |  |  |  |
| --- | --- | --- | --- | --- |
| int Skylark\_Clutch::st\_Developing | ( |  | ) |  |

protected

References Skylark\_Base::Age, ClutchMortProb, Cooling\_Rate\_Eggs, Skylark\_Base::DailyMortality(), EggTemp, TAnimal::m\_OurLandscape, Skylark\_Base::m\_OurPopulationManager, MD\_Hatch, MD\_Threshold, MinDegrees, Mother, Skylark\_Female::Supply\_NestTime(), Landscape::SupplyDayInYear(), Landscape::SupplyTemp(), CfgFloat::value(), and Skylark\_Population\_Manager::WriteSKPOM1().

Referenced by EndStep().

{

#ifdef \_\_CJTDebug\_5

if ( IsAlive() != 0x0DEADC0DE ) DEADCODEError();

#endif

if ( DailyMortality( ClutchMortProb ) ) {

#ifdef \_\_SKPOM

m\_OurPopulationManager->WriteSKPOM1( m\_OurLandscape->SupplyDayInYear(), 1001 );

#endif

return 2; //transfer to state die

}

if ( Age > 0 ) // No development on the first day when just laid

{

// Timing : This function is called once at the end of each day before

// the next day

// Develop according to ambient temperature, and how long the female

// is incubating for

int incubated\_time = Mother->Supply\_NestTime(); // this includes night

int off\_nest\_time = 24 \* 60 - incubated\_time;

//double Rain = m\_OurLandscape->SupplyRain();

//int Daylength = m\_OurLandscape->SupplyDaylength();

//m\_OurPopulationManager->WriteToTest2File(m\_OurLandscape->SupplyDayInYear(),off\_nest\_time);

// they start at EggTemp degrees and cool to 'cool'

double cool\_rate\_pmin = Cooling\_Rate\_Eggs / 60.0;

double Temp = m\_OurLandscape->SupplyTemp(); // 9.97 = COrrection for day/night temps in �tjylland

if ( Temp >= EggTemp ) Temp = EggTemp - 0.000001;

// If off\_nest\_time > daylength/2 then the birds are spending more time off the nest than on.

// in this case the trip length must go up

//double TripNumber=cfg\_FoodTripsPerDay.value();

double TripLength=cfg\_sk\_triplength.value();

/\*

if ( off\_nest\_time >= ( Daylength - 10 ) ) { // -10 allows us not to get a division by zero later

if ( ++m\_baddays > 5 ) {

#ifdef \_\_SKPOM

m\_OurPopulationManager->WriteSKPOM1( m\_OurLandscape->SupplyDayInYear(), 1003 );

#endif

return 2; //TransferToState(Die);

}

} else {

if ( off\_nest\_time > ( Daylength / 2.0 ) ) {

// getting cold, so assume a different strategy or maximising the number of on nest times of 10 minutes

int on\_nest = Daylength - off\_nest\_time;

TripNumber = on\_nest / 10.0;

TripLength = off\_nest\_time / TripNumber;

}

else {

TripLength = 10;

}

}

\*/

//

// We have the time incubated - but this includes re-warming time. So make

// the assumption that warming/cooling are linear and equal rates

// Now calculate the minimum temperature

// double cool = EggTemp - ( ( cool\_rate\_pmin \* TripLength ) \* ( ( EggTemp - Temp ) / 2.0 ) );

double cool = EggTemp - ( ( cool\_rate\_pmin \* TripLength ) \* ( ( EggTemp - Temp ) / 2.0 ) );

// This cannot be lower than ambient (There is a problem here since ambient

// temperature when the eggs are not incubated is higher than the mean used

// here because foraging is in the daytime

if ( cool < Temp ) cool = Temp;

// Now calculate how many minutes were spent cooling down to this temp (if

// cool is higher than Temp then cooltime should be the same as TripLength

// Next line to prevent overflow

double cooltime = ( EggTemp - cool ) / ( cool\_rate\_pmin \* ( ( EggTemp - Temp ) / 2.0 ) );

// Eggs will spend incubated time-cooltime at EggTemp, off\_nest\_time-cooltime

// at cool, and 2xcooltime at the mean of EggTemp and cool

double AverageOffNestTemp = ( cool + EggTemp ) / 2.0;

/\* if ( Rain > 5 ) { MinDegrees += ( ( 24 \* 60 ) - Daylength ) \* ( EggTemp - MD\_Threshold ); if ( m\_baddays++ > 5 ) {

m\_OurPopulationManager->WriteToTest2File( m\_OurLandscape->SupplyDayInYear(), 1009 );

return 2; //TransferToState(Die); } } else \*/

{

MinDegrees += (int) (( incubated\_time - ( cooltime \* 2.0 ) ) \* ( EggTemp - MD\_Threshold )); // took \*2 away

double offnesttemp = cool - MD\_Threshold;

if ( offnesttemp < 0 ) offnesttemp = 0; // No development, rather than minus

MinDegrees += int (( off\_nest\_time - cooltime ) \* ( offnesttemp ));

double transitiontemp = (int) AverageOffNestTemp - MD\_Threshold;

if ( transitiontemp < 0 ) transitiontemp = 0; // No development, rather than minus

MinDegrees += (int) (( 2.0 \* cooltime ) \* ( transitiontemp )); // was 1.0 - this change assumes immediate re-heating

}

/\* double scalingfactor = 1.0; int Daylength = m\_OurLandscape->SupplyDaylength( );

int daytimeonnest=Daylength-off\_nest\_time; double Rain = m\_OurLandscape->SupplyRain(); if (Rain >10) {

MinDegrees+=(24\*60)-Daylength; if (m\_baddays++>5) {

m\_OurPopulationManager->WriteToTest2File( m\_OurLandscape->SupplyDayInYear(), 1009 ); return 2; //TransferToState(Die); } }

else { if ((daytimeonnest \* scalingfactor) >= off\_nest\_time) { MinDegrees+=24\*60; // No problem, got all day } else {

if (daytimeonnest<=0) { MinDegrees+=incubated\_time; if (m\_baddays++>5) {

m\_OurPopulationManager->WriteToTest2File( m\_OurLandscape->SupplyDayInYear(), 1009 ); return 2; //TransferToState(Die);

}; // This counter just keeps going up until death } else { // Here we have used some time being cold, but not too much

int uncompensatedtime=off\_nest\_time-(daytimeonnest\*scalingfactor);

MinDegrees+=(24\*60)-uncompensatedtime; // Only part of the day } } } \*/

}

// Have we achieved enough minute degrees

if ( MinDegrees > MD\_Hatch ) {

#ifdef \_\_SKPOM

m\_OurPopulationManager->WriteSKPOM1(m\_OurLandscape->SupplyDayInYear(),Age);

#endif

return 1; //TransferToState(Hatch);

} else // if they reach the age of 18 they die if not hatched

if ( Age == 18 ) {

#ifdef \_\_SKPOM

m\_OurPopulationManager->WriteSKPOM1( m\_OurLandscape->SupplyDayInYear(), 1002 );

#endif

return 2; //TransferToState(Die);

} else {

Age++;

return 0;

}

}

|  |  |  |  |  |
| --- | --- | --- | --- | --- |
| void Skylark\_Clutch::st\_Dying | ( |  | ) |  |

protected

References TALMaSSObject::CurrentStateNo, Skylark\_Base::m\_CurrentSkState, Mother, Skylark\_Female::OnClutchDeath(), and toss\_Destroy.

Referenced by EndStep().

{

#ifdef \_\_CJTDebug\_5

if ( IsAlive() != 0x0DEADC0DE ) DEADCODEError();

#endif

// Timing: Zero time

// Only called before object is destroyed

if ( Mother ) Mother->OnClutchDeath();

m\_CurrentSkState = toss\_Destroy; // will kill it at end of step!

CurrentStateNo = -1;

}

|  |  |  |  |  |
| --- | --- | --- | --- | --- |
| int Skylark\_Clutch::st\_Hatching | ( |  | ) |  |

protected

References Skylark\_struct::bx, Skylark\_struct::by, Clutch\_Size, Skylark\_Population\_Manager::CreateObjects(), Nestling\_struct::Dad, Skylark\_struct::L, Skylark\_Base::m\_Born\_x, Skylark\_Base::m\_Born\_y, TAnimal::m\_Location\_x, TAnimal::m\_Location\_y, Skylark\_Base::m\_MyHome, TAnimal::m\_OurLandscape, Skylark\_Base::m\_OurPopulationManager, Skylark\_Base::m\_OurTerritories, Skylark\_struct::mh, Mother, Skylark\_Female::MyMate, Skylark\_Female::OnEggsHatch(), Skylark\_Adult::Paired, Skylark\_struct::Terrs, Landscape::Warn(), Skylark\_struct::x, and Skylark\_struct::y.

Referenced by EndStep().

{

#ifdef \_\_CJTDebug\_5

if ( IsAlive() != 0x0DEADC0DE ) DEADCODEError();

#endif

// Timing: Occurs at the end of a day (e.g. midnight-1 minute)

// Must create Clutch\_Size number of Skylark\_Hatchling Objects

// They tell Mother that they have been created when they exist in the system

Nestling\_struct \* Ch;

if ( Mother->Paired ) {

Ch = new Nestling\_struct;

Ch->Dad = Mother->MyMate; // Needs a pointer to dad not mum

Ch->x = m\_Location\_x;

Ch->y = m\_Location\_y;

Ch->L = m\_OurLandscape;

Ch->Terrs = m\_OurTerritories;

Ch->bx = m\_Born\_x;

Ch->by = m\_Born\_y;

Ch->mh = m\_MyHome;

#ifdef \_\_CJTDebug\_7

if ( Clutch\_Size == 0 ) {

g\_land->Warn( "Skylark\_Clutch::st\_Hatching(): DeBug7 ", NULL ); exit( 1 );

}

#endif

m\_OurPopulationManager->CreateObjects( 1, this, NULL, Ch, Clutch\_Size );

// object destroyed by m\_OurPopulationManager

delete Ch;

Mother->OnEggsHatch(); // Sets in motion feeding and she tells male

return 1;

} else {

return 0;

}

}

|  |  |  |  |  |
| --- | --- | --- | --- | --- |
| void Skylark\_Clutch::StartDeveloping | ( |  | ) |  |

inline

References toss\_Developing.

Referenced by Skylark\_Female::st\_Laying().

{

m\_CurrentSkState = toss\_Developing;

// Must tell the pop man how many eggs were made

m\_OurPopulationManager->incTotalEggs(Clutch\_Size);

}

|  |  |  |  |  |  |
| --- | --- | --- | --- | --- | --- |
| void Skylark\_Clutch::Step | ( | void |  | ) |  |

virtual

Reimplemented from TAnimal.

References Skylark\_Base::m\_CurrentSkState, Mother, TALMaSSObject::StepDone, Skylark\_Female::SupplyMyClutch(), toss\_CDying, toss\_Destroy, toss\_Developing, toss\_Hatching, toss\_Initiation, and Landscape::Warn().

{

#ifdef \_\_CJTDebug\_5

if ( IsAlive() != 0x0DEADC0DE ) DEADCODEError();

#endif

if ( StepDone || m\_CurrentSkState == toss\_Destroy ) return;

#ifdef \_\_CJTDebug\_5

if ( Mother ) {

if ( Mother->SupplyMyClutch() != this ) {

g\_land->Warn( "Skylark\_Step::Step(): Debug\_5 ", "" );

exit( 1 );

}

}

#endif

switch ( m\_CurrentSkState ) {

case toss\_Initiation: // Initial state. Will wait here until told by female that she is incubatig

StepDone = true;

m\_CurrentSkState = toss\_Initiation;

break;

case toss\_Developing:

StepDone = true;

break;

case toss\_Hatching:

StepDone = true;

break;

case toss\_CDying:

StepDone = true;

break;

default:

g\_land->Warn( "Skylark\_CLutch::Step(): Unknown State ", "" );

exit( 1 );

}

}

---

## Member Data Documentation

int Skylark\_Clutch::Clutch\_Size

Referenced by Skylark\_Clutch(), and st\_Hatching().

int Skylark\_Clutch::m\_baddays

protected

Referenced by Skylark\_Clutch().

int Skylark\_Clutch::MinDegrees

protected

Referenced by Skylark\_Clutch(), and st\_Developing().

Skylark\_Female\* Skylark\_Clutch::Mother

Referenced by OnMumGone(), Skylark\_Clutch(), st\_Developing(), st\_Dying(), st\_Hatching(), Step(), and Skylark\_Female::Step().

---

The documentation for this class was generated from the following files:

- skylarks\_all.h
- skylarks\_all.cpp


- Skylark\_Clutch
- Generated on Thu Jan 10 2013 13:15:37 for ALMaSS Skylark ODdox by
   1.8.1.1
